# The relationship between donor-recipient genetic distance and long-term kidney transplant outcome

**DOI:** 10.12688/hrbopenres.13021.1

**Published:** 2020-07-29

**Authors:** Caragh P. Stapleton, Graham M. Lord, UK and Ireland Renal Transplant Consortium, Peter J. Conlon, Gianpiero L. Cavalleri

**Affiliations:** 1 Department of Molecular and Cellular Therapeutics, Royal College of Surgeons in Ireland, Dublin, Ireland; 2 Faculty of Biology, Medicine and Health, University of Manchester, Manchester, UK; 3 NIHR Biomedical Research Centre at Guy’s and St Thomas’, NHS Foundation Trust and King’s College London, London, UK; 4 Department of Nephrology, Beaumont Hospital, Dublin, Dublin, Ireland; 5 Department of Medicine, Royal College of Surgeons in Ireland, Dublin, Ireland

**Keywords:** shared genetic ancestry, identity-by-state, identity-by-descent, transplant, kidney transplant, graft failure, HLA

## Abstract

**Background: **We set out to quantify shared genetic ancestry between unrelated kidney donor-recipient pairs and test it as a predictor of time to graft failure.

**Methods: **In a homogenous, unrelated, European cohort of deceased-donor kidney transplant pairs (n pairs = 1,808), we calculated, using common genetic variation, shared ancestry at the genic (n loci=40,053) and genomic level. We conducted a sub-analysis focused on transmembrane protein coding genes (n transcripts=8,637) and attempted replication of a previously published nonsynonymous transmembrane mismatch score. Measures of shared genetic ancestry were tested in a survival model against time to death-censored graft failure.

**Results: **Shared ancestry calculated across the human leukocyte antigen (HLA) significantly associated with graft survival in individuals who had a high serological mismatch (n pairs = 186) with those who did not have any HLA mismatches indicating that shared ancestry calculated specific loci can capture known associations with genes impacting graft outcome. None of the other measures of shared ancestry at a genic level, genome-wide scale, transmembrane subset or nonsynonymous transmembrane mismatch score analysis were significant predictors of time to graft failure.

**Conclusions: **In a large unrelated, deceased-donor European ancestry renal transplant cohort, shared donor-recipient genetic ancestry, calculated using common genetic variation, has limited value in predicting transplant outcome both on a genomic scale and at a genic level (other than at the HLA loci).

## List of Abbreviations

eGFR, estimated glomerular filtration rate; HLA, human leukocyte antigen; IBD, identity by descent; IBS, identity by state; SNP, single nucleotide polymorphism; UKIRTC, United Kingdom and Ireland Renal Transplant Consortium; GWAS, Genome-wide association study; PCA, principal components analysis.

## Introduction

Organ allocation is a major issue in the field of transplantation. It is vital that organs are matched in a way that facilitates the best possible outcome for the recipient and for the transplanted organ, so that the organ goes to the person for whom it will last the longest. For many decades, renal transplants have been allocated largely on the basis of preferential HLA scoring, with reports dating back as far as 1965 showing the impact of HLA matching on graft outcome ^1, 2^. A perfect HLA “match” does not guarantee a successful outcome and in individuals with zero HLA mismatches, acute rejection and early graft failure are still present ^3^. This indicates that other, potentially genetic, factors influence graft outcome and the success of the transplant.

There are large differences in graft survival between ethnicities, with African Americans having the poorest graft survival rates ^4^. However, it is unclear to what extent these differences are attributable to genetic, socioeconomic, and/or behavioural factors ^5, 6^. A recent study of donor and recipient kidney transplant pairs found that ethnically matched transplants had significantly better graft survival, when compared to non-ethnically matched pairings. However this significance disappeared when adjustment was made for clinical variables including HLA mismatch, sensitization, sex, age and cold ischaemic time ^7^. Thus, the impact of shared ancestry between the kidney donor and recipient on graft outcome remains unclear.

Recent studies have developed distance scores based on exome sequencing data which are reported to predict graft outcome. For example, one study created an allogenomic mismatch score ^8^ using non-synonymous variants from transmembrane protein genes. In a cohort of 53 donor-recipient kidney transplant pairs of admixed ethnicity (including 32% African ancestry individuals), this score was significantly associated with estimated glomerular filtration rate (eGFR), after correcting for HLA mismatches (at A, B and DR), donor age and time of eGFR measurement post-transplant. A similar mismatch study based on non-synonymous variation in non-HLA transmembrane and secreted protein-encoding genes also found that a greater level of mismatch between the donor and recipient associated with reduced graft survival ^9^.

The explosion of dense genotype single nucleotide polymorphism (SNP) data has allowed for the accurate measure of shared ancestry at the individual and also population level. There are a number of methods for quantifying shared ancestry including haplotype “painting” ^10^, identity-by-state (IBS) and identify-by-descent (IBD) measures.

In this study, we set out to investigate the impact of shared genetic ancestry between donor-recipient transplant pairs, on graft outcome. We quantified shared genetic ancestry at the genomic level, genic level and across a specific subset of transmembrane protein encoding genes and tested these shared ancestry measures as a predictor of time to graft failure in a population of unrelated, European, deceased-donor kidney transplant pairs.

## Methods

### Cohort

Kidney donor and recipient pairs (n=2,094) were recruited via the United Kingdom and Ireland Renal Transplant Consortium (UKIRTC) ^11^. All transplants were deceased donor grafts and paired on the basis of preferential HLA matching and recipient waiting time. Transplants in the UKIRTC cohort took place between 1981–2007. Only data on the first transplant was considered. All individuals were GWAS genotyped as part of the UKIRTC and the Wellcome Trust Case Control Consortium 3 (see 11 for further details).

The following inclusion/exclusion criteria were applied: participants must have 1) been of European ancestry (determined via principal components analysis (PCA) – see *Extended data*, Supplementary methods for details) ^12^, 2) have donated/received a renal transplant and 3) be unrelated to level of 3 ^rd^ degree relative (PIHAT<0.1 calculated using PLINK v1.9 ^13, 14^).

Clinical predictors of graft survival were tested using STATA v13 in a Cox proportional hazards model and significant variables were included in the analysis as covariates. Acute rejection status was available for 1,099 individuals in our cohort. See *Extended data*, Supplementary methods ^12^ for details on imputation. Details on HLA typing were published elsewhere ^11^.

### Ethics approval and consent to participate

Ethics approval was granted by the Hammersmith and Queen Charlotte's & Chelsea Research Ethics Committee REC No 08/H0707/1.

### Genome-wide shared ancestry

We quantified shared genetic ancestry between donors and recipients at a genome-wide scale using three methods.

***Method 1: Identity by descent (IBD)***. IBD here refers to the amount of shared genetic material between two individuals that is inherited from a recent common ancestor. We quantified the proportion of the genome that is IBD between the donor-recipient pairs using the PIHAT function in PLINK ^13, 14^. The data was pruned for linkage disequilibrium using a 1000-kb window size, 100-kb step size and r ^2^ threshold of 0.2.

***Method 2: Identity by state (IBS)***. IBS quantifies the number of shared alleles between two individuals. Alleles that are IBS can be inherited from a recent common ancestor or shared by chance. We quantified the proportion of shared alleles across the genome between each donor and recipient pair using the DST measure in PLINK ^13, 14^. Data was pruned for linkage disequilibrium following the same criteria as outlined above for the IBD method.

***Method 3: Chromopainter***. We created a mosaic of an individual’s genome from the haplotypes of all the other individuals in the dataset using Chromopainter ^10^. We calculated the matrix of donated haplotype lengths and associated haplotype-donors, for each individual in our dataset (i.e. the “chromolength” output from Chromopainter). The correlation between the chromolength chunks (donated from each individual in our dataset) of the donor and recipient in a given donor/recipient pair was calculated using the “corr” function in Microsoft Excel (with a perfectly matched mosaic genome having a score of 1). The correlation was then used as the measure of shared genetic ancestry.

These three measures of shared ancestry were tested against time to graft failure using a Cox Proportional Hazards Model using the R survival package (version 2.41–3) and the cox.ph function ^15, 16^. We analysed two different survival analysis models; in the ‘full’ model we included significant clinical predictors as covariates, in the ‘baseline’ model we excluded clinical covariates.

### Per genic region analyses

We examined whether shared ancestry between donor and recipient pairs at any specific gene, calculated using the IBD PIHAT and IBS DST methods, was associated with graft survival. Hg19 (human genome assembly 19) coordinates of protein-coding loci were obtained using the UCSC Genome Browser Table Browser via the “Genes and Predictions” group on the USCS gene track ^17^. The coordinates with a 50-kb buffer region added were used to extract the imputed genotype information for each individual in each donor recipient pair using PLINK, creating a file per genic locus. The files were pruned for LD in PLINK (“–indep-pairwise” command) using 1000-kb window size, 100-kb step-size and r ^2^ threshold of 0.2. The PIHAT and DST scores were calculated for each of the thinned genic region files using PLINK’s --genome command. The PIHAT and DST scores for each gene were then extracted for each donor recipient pair.

PIHAT and DST scores for each gene was tested against time to graft failure, adjusting for significant clinical covariates, using R’s survival package ^15^. We corrected for multiple testing via the Bonferroni method. The total number of loci tested was 40,053 but greater than 50% of these were transcripts coded from overlapping regions and so we only considered independent loci (n= 16,765) when correcting for multiple testing.

As sanity check for the genic approach, we assessed whether genic shared ancestry measures could detect the known effect of the HLA on transplant outcome. As the UKIRTC dataset is composed of donor recipient pairs who were paired by preferential HLA matching and thus skewed towards pairs with fewer mismatches, we focused on the subset of transplant pairs with 0, 5 or 6 serological HLA mismatches. Time to death censored graft failure was tested against the total serological HLA mismatch measure, and against the IBD and IBS measures, in a Cox proportional hazards model using R. As this is a replication of the HLA mismatch measures, we did not apply Bonferroni correction and used a standard significance threshold of p=0.05.

### Shared ancestry across transmembrane protein coding genes

As a subgroup analysis, we focused on the scores for transmembrane protein coding genes specifically, calculated in donor-recipient pairs using the IBD PIHAT and IBS DST measures (see methods section 5.2). The list of transmembrane protein coding was obtained using the same methods described previously ^9^. PIHAT and DST scores were extracted for these loci from the per-genic region results (see methods section 5.3). The PIHAT score was summed across all of the transmembrane genes to create an overall score. This transmembrane PIHAT score was then normalized, so the mean was 0 and standard deviation was 1. The same process was repeated for DST. The normalized transmembrane DST and PIHAT scores were then tested against time to graft failure in a Cox proportional hazards model using the survival package in R. Models were adjusted for significant clinical predictors of time to graft failure (see *Extended data*, Supplementary Table 1 ^12^).

We also analysed the same set of transmembrane genes applying a univariate approach, where the PIHAT/DST calculated for each gene was considered separately and tested against time to graft failure as per the previous paragraph. To correct for multiple testing within this sub-group analysis, each gene was corrected for as opposed to each transcript (as some genes had multiple overlapping transcripts). In total, we corrected for 4,873 tests.

Finally, we carried out an exact replication analysis of the nonsynonymous transmembrane mismatch score which was recently reported to predict time to graft failure ^9^. Following the protocol as described by Reindl-Schwaighofer *et al.* ^9^, we imputed the HLA eplet mismatches using HLA matchmaker. Next, using VEP we annotated the imputed genotypes. We used the DR-mismatch program ^9^ to calculate the nonsynonymous transmembrane mismatch score. We also calculated the overall genotype mismatch score and the overall nonsynonymous mismatch score (i.e. not limited to transmembrane protein encoding genes). Matching the parameters of the analysis presented in 9, we censored graft survival to 10 years post-transplant and excluded individuals with less than 3 months of follow-up post-transplant (see *Extended data*, Supplementary Table 5 ^12^ for cohort description). We carried out a Cox proportional hazards model against time to death censored graft failure of the interquartile range of the nonsynonymous transmembrane mismatch score including genome-wide mismatch score, genome-wide nonsynonymous mismatch score, HLA serological mismatch, HLA eplet mismatch, donor age, donor sex, transplant centre and IBD (using PIHAT score as measure). We also tested the nonsynonymous transmembrane mismatch score in a univariate model which was not adjusted for additional covariates.

## Results

Graft failure data was available for 1,808 donor-recipient pairs in our cohort with a total of 399 failure events (death censored). The average time to death censored graft failure was 6.04 years (range, 37–9,021 days) and average follow-up in those without a failure event was 8.87 years (range, 89–9,038 days). In order to determine the influence of clinical covariates on time to graft failure in the UKIRTC dataset, we applied a Cox proportional hazards model (see *Extended data*, Supplementary Table 1 ^12^). Donor age and acute rejection at 1 year emerged from the Cox model as significant predictors of graft failure and therefore were included as covariates in our shared ancestry survival analysis.

### Genome-wide shared ancestry

We set out to test the effect of the three different genome-wide shared ancestry measures (IBS, IBD and Chromopainter) on time to death censored graft failure using a Cox proportional hazard model.

These shared ancestry measures were successfully calculated on 1,815 donor-recipient pairs (see *Extended data*, Supplementary Figure 1 ^12^ for distributions of Chromopainter, IBD and IBS measures). Of these pairs, 927 had no detectable IBD relationship (i.e. PIHAT = 0) with 888 pairs having a PIHAT greater than 0. Due to the large number of pairs with PIHAT = 0, we carried out subsequent analyses on two groups; 1) those pairs who shared an IBD of less than or equal to 3 ^rd^ degree relative, excluding those with no detected IBD and 2) pairs with an IBD of less than or equal to 3 ^rd^ degree relative, including those with no detected IBD (see *Extended data*, Supplementary Table 2 ^12^).

These shared ancestry measures were then tested against time to graft failure in a Cox proportional hazards model. None of the genome-wide shared ancestry measures were significant in our full (with clinical covariates) or baseline models (without clinical covariates, see Table 1).

**Table 1.** Genome-wide shared ancestry measures in time to graft failure Cox proportional Hazards model. Full model was adjusted for donor age and acute rejection (within 12 months post-transplant). Baseline model was not adjusted for additional covariates.

	Model	HR	lower	upper	P	N	N events
**Chromopainter corr**	full	5	0.28	89.58	0.27	1099	179
**PIHAT**	full	1.50E-08	2.40E-20	8693.76	0.19	1099	179
**PIHAT - Zero =NA**	full	1.40E-13	2.70E-32	750000	0.18	512	82
**DST**	full	1.20E-16	2.30E-62	5.90E+29	0.49	1099	179
**Chromopainter corr**	baseline	4.64	0.78	27.52	0.09	1808	399
**PIHAT**	baseline	0.02	5.90E-10	599466	0.65	1808	399
**PIHAT - Zero =NA**	baseline	2.00E-08	4.30E-20	9457	0.2	885	202
**DST**	baseline	0.009	4.20E-35	1.70E+30	0.9	1808	399

HR, hazard ratio; 95% CI, upper and lower 95% confidence intervals; P, uncorrected p-value. PIHAT - Zero =NA, any donor recipient pair with a PIHAT score of 0 was removed from this analysis; Chromopainter corr, chromopainter correlation measure; N, number of donor-recipient pairs included in the analysis (i.e. had full clinical covariate data and PIHAT/DST scores available).

### Shared ancestry per genic region results

Given the lack of correlation between graft outcome and donor-recipient genome-wide-level shared ancestry measures, we next tested if the shared ancestry between a donor and recipient at any one particular gene could predict graft outcome.

**Figure 1.**
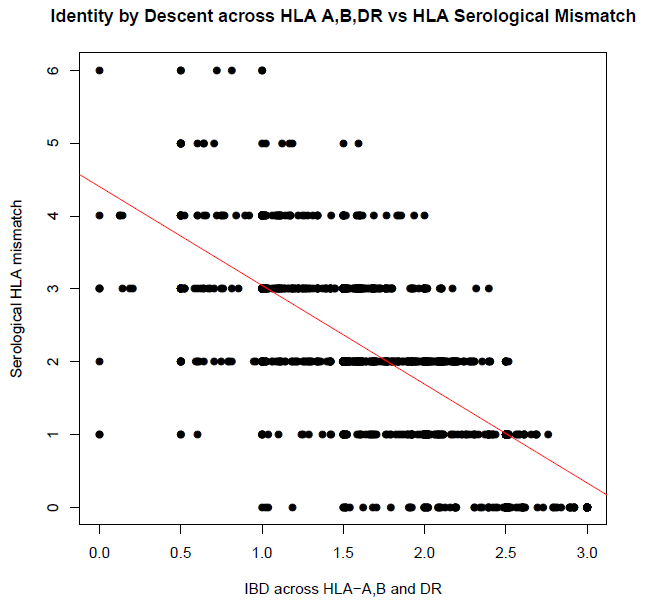
Correlation between total HLA mismatches and PIHAT across HLA (p = 3.57x10 ^-223^ and r ^2^ of 0.48). Total mismatches were calculated across HLA-A, B and DR using typing by serology. PIHAT (measure of IBD) was calculated for HLA-A, B and DR individual and combined by summing the PIHAT scores for each locus.

**Figure 2.**
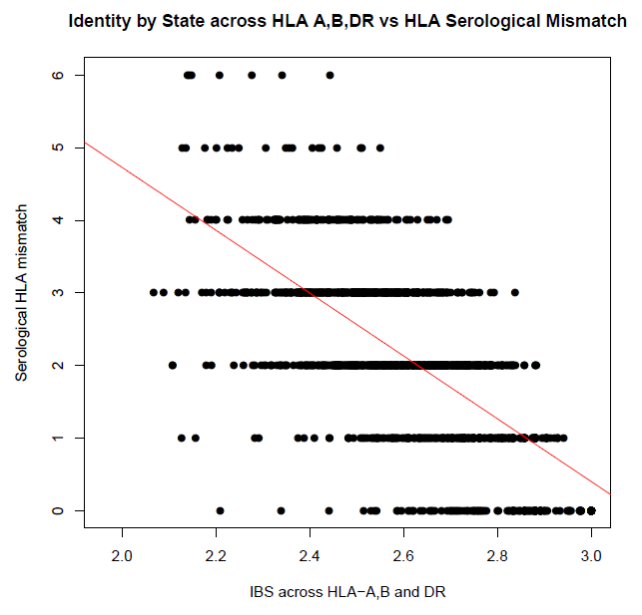
Correlation between total HLA mismatches and DST across HLA (p= 5.74x10 ^-222^ and r ^2^ = 0.47). Total mismatches were calculated across HLA-A, B and DR using typing by serology. DST (measure of IBS) was calculated for HLA-A, B and DR individual and combined by summing the DST scores for each loci.

The HLA is a known predictor of graft outcome. To determine if our shared ancestry measures could reproduce the known effect of the HLA on graft outcome, we tested the correlation between IBD and IBS scores calculated across the HLA region, and the clinically reported combined serological HLA mismatch score (at HLA-A, B and DR). Both IBD and IBS were highly significantly correlated with total serological mismatch scores (P = 3.57x10 ^-223^/r ^2^ = 0.48 and 5.74x10 ^-222^/0.47, respectively (see Figure 1 and Figure 2).

As donor recipient pairs in the UKIRTC were selected on the basis of preferential HLA matching, the data was skewed towards having a low number of HLA mismatches. In this context, we selected donor-recipient pairs who had either zero-, or 5/6 HLA mismatches for regression analyses testing HLA distance with graft outcome. In this subset of donor-recipient pairs, we found that the total serologically typed HLA mismatch was associated time to graft failure (p=0.009, N=186, N events = 40, HR = 1.22, SE = 0.08).

Having reproduced the known predictive ability of HLA serological scores, we next compared, in the same group of individuals, the IBD and IBS HLA scores, to time to graft failure in a Cox proportional Hazards model. IBD and IBS scores were both significantly associated with time to graft failure with more shared IBD, or a greater amount of IBS associated with a longer time to graft failure (see Table 2). When we conditioned on serological HLA matches, the IBD/IBS signals were no longer significant, suggesting that the IBD/IBS scores are capturing similar variability as the serological typing.

**Table 2.** Cox proportional hazards results: DST and PIHAT HLA measures vs time to graft failure.

	HR	Lower CI	Upper CI	P	N	N events
**PIHAT HLA A, B, DR**	0.65	0.43	0.98	0.04	186	40
**DST HLA A, B, DR**	0.29	0.1	0.89	0.03	186	40

Outcome, time to graft failure; PIHAT HLA A, B, DR, PIHAT/DST between the given donor and recipient calculated across HLA A, B and DR loci; HR, hazards ratio; CI, 95% confidence interval; p, p-value; N, number of donor-recipient pairs included in the analysis; N events, number of failure events.

The concordance statistic measures the proportion of pairs of individuals in which the individual with the higher-risk predictor had a failure event before the individual with the lower-risk predictor. We tested this statistic for the serological HLA score and the IBD/IBS scores (serological mismatch concordance = 0.56, standard error (SE) = 0.03; IBS across HLA concordance = 0.56, SE =0.05; IBD across HLA concordance = 0.57, SE = 0.05). This indicates that IBS/IBD per gene measures can capture known associations with genes impacting graft outcome.

We then went on to test shared ancestry in individual genes across the entire genome. We compared the IBS and IBD measures calculated from each coding region in the genome (that contained at least one SNP after quality control measures were applied) to the afore-mentioned outcome measures. No gene reached genome-wide significance, for either the IBD or IBS measures (see *Extended data*, Supplementary tables 9–12 ^12^).

The HLA-A (IBD p = 0.017 and IBS p = 0.045) and DR (IBD p = 0.009 and IBS p = 0.01) loci came out in the top 5% of the distribution for the time to graft failure analysis, but did not survive correction for multiple testing (see Supplementary table 3).

We also examined the per gene IBD and IBS scores in a baseline model which excluded clinical covariates. No single gene survived correction for multiple testing (n = 1,804, n events = 375; see *Extended data*, Supplementary tables 11–12 ^12^).

### Shared ancestry across transmembrane protein encoding genes results

Given previously published analysis ^8, 9^ showing a significant association between donor and recipient allelic mismatches across genes encoding transmembrane proteins and graft outcome, we tested the association between the amount of shared genetic ancestry calculated across these transmembrane proteins and time to death censored graft survival. Neither the normalized transmembrane IBD nor IBS score were a significant predictor of time to graft failure even before correction for multiple testing in our baseline (IBD p = 0.50, IBS p=0.35, n = 1,804, n events = 395) or full models (n= 1030, n events = 160, see *Extended data*, Supplementary tables 13–17 ^12^).

Transmembrane genes were also analysed in a univariate model, where the IBD/IBS measure for each transmembrane gene was tested against time to graft failure. None of the transmembrane genes reached significance after correcting for multiple testing (corrected for 4783 tests, see *Extended data*, Supplementary tables 13 –17 ^12^).

Finally, we went on to attempt to replicate the findings of Reindl-Schwaighofer *et al.* ^9^, who demonstrated a significant correlation between a transmembrane nonsynonymous donor-recipient mismatch score and time to ten-year and death censored graft failure. We did not find a significant correlation between in allogenomic mismatch score and time to graft failure in the univariate or multivariate (adjusted for additional covariates - see *Methods* section) models (see *Extended data*, Supplementary tables 7 and 8 ^12^ for results).

## Discussion

Our findings show that shared donor-recipient genetic ancestry, beyond 3 ^rd^ degree relative, has limited value in predicting transplant outcome both on a genomic scale and at a genic level in deceased donor European ancestry renal transplant pairs. Our findings also suggest that IBD and IBS-based distance methods can be used to capture serological mismatch scores, and show comparative predictive ability.

Our analysis of shared ancestry at the genome-wide level indicates that, in a European-descent homogenous population, the level of shared ancestry between unrelated (defined here as <3 ^rd^ degree relative) deceased-donor and recipient pairs is not a clinically relevant predictor of graft survival. This is an important finding as it suggests that when choosing between deceased donors and recipients and where all of North-West European ancestral background, the level of shared ancestry (beyond 3 ^rd^ degree) between donor and recipient is irrelevant.

This study was carried out on a homogenous North West European ancestry population and therefore should not be generalized to admixed, or populations of different ancestry. Further work is required to determine if shared ancestry between donors and recipients impacts graft survival when the levels of shared ancestry has greater tendency for fluctuation within a given population, in particular admixed populations (for example, in African American communities).

We did not find any single gene that significantly associated with time to graft failure. This result indicates that, beyond the HLA, single gene shared-ancestry effects do not have a clinically relevant impact on graft survival. Notably, the IBD and IBS measures calculated at HLA-A and DR were found in the top 5% of genes most significantly associated with time to graft failure, but did not reach genome-wide significance. However, we found an effect of shared ancestry calculated across the HLA when we compared highly mismatched individuals for HLA serological typing, to those who were perfectly matched. We also showed a highly significant correlation between the total number of HLA serological mismatches and the amount of shared genetic ancestry calculated across the same HLA loci (A, B and DR). This demonstrates that these shared ancestry measures can pick up clinically relevant genetic differences between donors and recipients, and indeed have potential as a proxy for serological testing. This is in line with previous studies that showed known associations with the HLA region can be picked up using IBD measures ^18, 19^. It is probable the HLA genes would have reached genome-wide significance in our genic analysis, if we had larger numbers of donor: recipient pairs and/or a cohort of transplant donors and recipients which had not been paired on the basis of preferential HLA matching.

Neither IBD nor IBS measures calculated across transmembrane protein coding loci were predictive of graft survival in our analysis. A previous exome mismatch study ^8^ and genome-wide association study (GWAS) based analysis ^9^ found that a score created via the quantification of specifically non-synonymous allelic mismatches at transmembrane and secreted proteins significantly predicted graft outcome whereas our shared ancestry measures considered all variation (i.e. including synonymous variation). We attempted to replicate the findings of the GWAS-based transmembrane nonsynonymous mismatch score ^9^; we did not see a significant correlation between the score and time to death censored graft survival with or without adjustment for additional covariates. However, we did see the same direction of effect as the original study. Potentially, differences between the cohort in this study and the original study may explain the lack of significance in our analysis. Our cohort was an older era and so was exposed to different immunosuppressants compared to that in the original study which was a more contemporary cohort. Further analysis with additional patient cohorts is required to determine whether this mismatch score will replicate in cohorts beyond that in the original report. The previous exome study ^8^ captured both common and rare variation, whereas our study considered common variation only. It is also possible and indeed likely that advancements in immunosuppressants are suppressing the effect of shared ancestry.

## Conclusions

In a large deceased-donor European ancestry renal transplant cohort, the amount of shared genetic ancestry between a donor and recipient had limited value in predicting transplant outcome both on the genome scale and at a genic level. However, the distance measures described in this study could describe a similar amount of the variability that is captured through serological HLA typing, and so have potential as an alternative to serological testing. Although we did not detect an effect of shared ancestry on graft outcome, studies capturing rare variation as well as studies examining admixed populations may uncover an important role of shared genetic ancestry on graft outcomes.

## Data availability

### Underlying data

The data used and/or analysed in the presented study was GWAS genotype chip array data and clinical data collected as part of the UKIRTC. Due to the sensitivity of the clinical patient data and genotype data used in this study, we are unable to share the data publicly. Access can be requested by researchers at accredited institutions. Access to data can be requested by emailing Peter J. Conlon ( peterconlon@beaumont.ie) or Gianpiero L. Cavalleri ( gcavalleri@rcsi.ie).

### Extended data

Harvard Dataverse: Supplementary Information: The relationship between donor-recipient genetic distance and long-term kidney transplant outcome. https://doi.org/10.7910/DVN/ZXAXTN ^12^.

File ‘Supplementary Information - shared ancestry manuscript’ (DOCX) contains the following extended data:

- Affiliations for UKIRTC consortium members
- Supplementary methods
- Supplementary Tables 1–8
- Supplementary Figure 1

Supplementary Tables 9–16 are available as individual DOCX files as part of the repository project.

## Authors' contributions

CPS, GLC and PJC designed the analyses. CPS carried out the data analyses and wrote the manuscript. GLC and PJC supervised the data analysis and manuscript writing. GML coordinated the data collection and genotyping of the UKIRTC dataset. The UKIRTC members carried out the patient recruitment, clinical data collection and provided DNA samples/genotype information for patients. All authors reviewed and provided feedback for the manuscript.
