# Supplementary material for: The relationship between donor-recipient genetic distance and long-term kidney transplant outcome
Source: HRB Open Res. 2020 Jul 29;3:47. [Version 1] doi: 10.12688/hrbopenres.13021.1 (PMC7888353; doi:10.12688/hrbopenres.13021.1)
Supplement: Peer review history

# Peer review: Reviewer response for version 1

**DOI:** 10.21956/hrbopenres.14114.r28469

**Published:** 2021-03-01

**Authors:** Madhav Menon

**Affiliations:** 1 Division of Nephrology, Department of Medicine, Recanati-Miller transplant Institute, Icahn School of Medicine at Mount Sinai, New York, NY, USA; 2 Nephrology, Yale School of Medicine, New Haven, CT, USA

Stapleton and colleagues use data on Deceased donor transplants from the UK/IR transplant registry to show that genome wide IBS/IBD do not independently associate with graft failure. They also do not find prior association with Austrian cohort data regarding total non Synonymous mismatches in genes predicted in a transmembrane or secreted location.

 These are important data to report from this homogenous north west European cohort. These findings are different from Reindl-Schwaighofer *et al, and Zhang et al, 2020 (not referenced - *should be appropriately referenced) ^1^.

- The homogeneity of the patient cohort used here could be depicted using a PCA plot based on the 1000 genome data. This would show difference between this and Zhang *et al.* report which was a clearly admixed cohort.
- An IBD distribution plot will also help visualize this.
- Details of the genotyping method used i.e. SNP chip etc. are missing. Were SNP chip versions different across data from this large /multicenter cohort?
- Was imputation performed? Details of pipeline used should be provided?
- Why were higher levels of relatedness excluded? Including them could still bring out a role for IBD that is greater than HLA mismatches?
- Is HLA serological mismatch used here? Both prior reports used PCR based mismatches. While PCR based data is also referenced later - it is unclear. Could have led to differences in findings.
- In the correlation with HLA mismatch levels and within group comparisons the 0 vs 5/6 is shown. The role of IBS/IBD in other levels of mismatches should be tested. In these intermediate levels a greater role may be identified.

Overall valuable data to report.

Is the work clearly and accurately presented and does it cite the current literature?

No

If applicable, is the statistical analysis and its interpretation appropriate?

Yes

Are all the source data underlying the results available to ensure full reproducibility?

Yes

Is the study design appropriate and is the work technically sound?

Yes

Are the conclusions drawn adequately supported by the results?

Yes

Are sufficient details of methods and analysis provided to allow replication by others?

Yes

Reviewer Expertise:

Kidney transplantation, long term graft damage, Donor recipient genetics.

I confirm that I have read this submission and believe that I have an appropriate level of expertise to confirm that it is of an acceptable scientific standard.

## References

[1] : Genome-wide non-HLA donor-recipient genetic differences influence renal allograft survival via early allograft fibrosis. *Kidney Int*.98(3) : 10.1016/j.kint.2020.04.039 758-768 10.1016/j.kint.2020.04.039 32454123PMC7483801

# Peer review: Reviewer response for version 1

**DOI:** 10.21956/hrbopenres.14114.r28649

**Published:** 2021-02-16

**Authors:** Krzysztof Kiryluk

**Affiliations:** 1 Department of Medicine, Division of Nephrology, Columbia University, College of Physicians & Surgeons, New York, New York, USA

This is an interesting study that tests the role of genetic similarity between kidney donors and recipients in predicting long-term allograft outcomes. The study has notable strengths, including a large number of genotyped donor-recipient pairs used in the analysis and a comprehensive set of genetic similarity metrics tested as potential predictors of allograft survival. The methods are well described and the statistical models appear appropriate. The results were largely negative, leading to the conclusion that the degree of DR ancestry sharing (with the exception of the HLA region) was not significantly associated with time to allograft failure. The authors appropriately discuss the fact that these findings were based on a homogenous European cohort, and might not be generalizable to more diverse or admixed populations. Overall, the paper is informative and the negative results do provide a meaningful contribution.

 I do have few minor suggestions that could improve the manuscript:

1. Given that this study produced largely negative results, it would be important to provide a power analysis (estimate the probability of type II error) and discuss the range of detectable effect sizes given the cohort size, the available follow up time, and the observed event rates. 
2. The main hypothesis of the study is that genetic donor-recipient dissimilarity would increase the risk of allograft failure by increasing the risk of rejection. It would therefore be informative to also include the analysis of rejection (any type), since rejection represents a more proximal outcome, and the study may have a better power to test for this association.
3. I am not entirely clear why the full model presented in Table 1 is adjusted for rejection events, since presumably rejection would mediate the effects on allograft survival. This model should probably be adjusted for HLA mismatch instead of rejection (to test for the effects of genetic mismatch that is independent of HLA, which I think would be a more meaningful hypothesis).
4. It would also be informative to see the following in the supplement: (1) histograms for the distributions of the key DR ancestry similarity metrics tested as predictors in the study, (2) PCA plot for the DR cohort in relationship to major continental ancestries, (3) QQ-plots for gene-based similarity tests. Please add HGNC-approved gene symbols to the Supplemental Tables. I would also consider replacing the term “genic regions” with “gene regions”. 

Is the work clearly and accurately presented and does it cite the current literature?

Yes

If applicable, is the statistical analysis and its interpretation appropriate?

Yes

Are all the source data underlying the results available to ensure full reproducibility?

Partly

Is the study design appropriate and is the work technically sound?

Yes

Are the conclusions drawn adequately supported by the results?

Yes

Are sufficient details of methods and analysis provided to allow replication by others?

Yes

Reviewer Expertise:

Human genetics.

I confirm that I have read this submission and believe that I have an appropriate level of expertise to confirm that it is of an acceptable scientific standard.

# Peer review: Reviewer response for version 1

**DOI:** 10.21956/hrbopenres.14114.r27907

**Published:** 2020-09-10

**Authors:** Adrian Dockery

**Affiliations:** 1 The School of Genetics & Microbiology, Trinity College Dublin, Dublin, Ireland

Dr. Stapleton and colleagues have produced a well-structured, well-written and refreshingly honest overview of genetic analyses undertaken to assess the relationship of shared genetic ancestry and time to graft failure in kidney donor recipients. 

 Sufficient background information relevant to the study has been provided. The methods are thoroughly described and potential limitations of the analyses are made very clear.

 The results are depicted clearly and discussed in a very balanced manner.

Is the work clearly and accurately presented and does it cite the current literature?

Yes

If applicable, is the statistical analysis and its interpretation appropriate?

I cannot comment. A qualified statistician is required.

Are all the source data underlying the results available to ensure full reproducibility?

Partly

Is the study design appropriate and is the work technically sound?

Yes

Are the conclusions drawn adequately supported by the results?

Yes

Are sufficient details of methods and analysis provided to allow replication by others?

Yes

Reviewer Expertise:

Clinical genetics

I confirm that I have read this submission and believe that I have an appropriate level of expertise to confirm that it is of an acceptable scientific standard.
